# Heme oxygenase‐1 induction mediates chemoresistance of breast cancer cells to pharmorubicin by promoting autophagy via PI3K/Akt pathway

**DOI:** 10.1111/jcmm.13800

**Published:** 2018-09-14

**Authors:** Lei Pei, Yirong Kong, Changfeng Shao, Xiao Yue, Zongling Wang, Na Zhang

**Affiliations:** ^1^ Department of General Surgery The People's Hospital of Pingyi County Pingyi Shangdong China; ^2^ Department of the Clinical Laboratory Qingdao Municipal Hospital Qingdao Shandong China; ^3^ Department of Transfusion The Affiliated Hospital of Qingdao University Qingdao Shandong China; ^4^ Department of Dermatology The Affiliated Hospital of Qingdao University Qingdao Shandong China

**Keywords:** autophagy, breast cancer cells, drug resistance, pharmorubicin

## Abstract

**Background:**

Concerns about breast cancer had become the most dangerous cancer to women over the world, more and more anti‐cancer agents are developed to treat this malignancy. Pharmorubicin is a cytotoxic drug, widely used in the treatment of breast cancer, but its role is limited because of chemoresistance produced by cells. This study focused on exploring the influence of autophagy on the resistance of pharmorubicin in breast cancer cells.

**Methods:**

The cell survival of breast cancer cells was detected by MTT. The mRNA expression of heme oxygenase‐1 (HO‐1) was tested by qRT‐PCR. The protein expression of HO‐1, autophagic proteins (LC3‐I,LC3‐II and Beclin‐1), PI3K and Akt was detected by Western blot. Cell autophagy was examined by Cyto‐ID Autophagy Detection Kit.

**Results:**

After being treated with pharmorubicin, the expression of HO‐1 and autophagy related proteins was significantly enhanced, but the cell survival ratio in the two cell lines decreased. After autophagy was inhibited, HO‐1 expression in two cells was down‐regulated. When pharmorubicin‐resistant cells were transfected with si‐HO‐1, the cell survival decreased and the protein expression of HO‐1, autophagic proteins (LC3‐II/LC3‐I and Beclin‐1) as well as autophagy were all down‐regulated, while in pharmorubicin‐resistant cells transfected with pcDNA3.1‐HO‐1, the results were reverse. When the PI3K or Akt was inhibited, PI3K, p‐Akt, HO‐1, autophagic proteins and autophagy were decreased remarkably.

**Conclusion:**

It was proved that HO‐1 induction mediated chemoresistance of pharmorubicin in breast cancer cells by promoting autophagy via PI3K/Akt pathway.

## 1 INTRODUCTION

Breast cancer is the second leading inducement of cancer death in women worldwide.^1^ Globally, it is estimated that 1.2 million women are newly diagnosed with breast cancer in 2016 and 0.5 million die from it each year.^2^ This malignancy represents a divergent group of tumours with characteristic molecular features, prognosis and the responses to current therapy.^3^ Pharmorubicin is a kind of effective therapy for breast cancer, and it can be used alone or combined with chemotherapy to reduce disease progression and has more favourable impact on survival of patients. Chemoresistance is considered a major factor influencing breast cancer clinical outcomes. However, current progress in finding a potent, selective method to overcome cancer drug resistance has become an urgent issue around the world.^1^

Heme oxygenase (HO) which is the limited enzyme in heme degradation catalyses the oxidation of heme to produce some biological active molecules: carbon monoxide, ferrous ion and biliverdin. HO has 2 isozymes, heme oxygenase‐1 (HO‐1) and heme oxygenase‐2 (HO‐2), and HO‐1 is high inductivity under various chemical and physical cellular stresses. Given that HO‐1 has cytoprotective properties, it has attracted great attention on promoting tumour cell survival in many cancers as followings: increased expression of HO‐1 promoted primary and metastatic prostate tumour growth^4^; Up‐regulating of HO‐1 expression might be effective in controlling cells migration in lung cancer^5^; HO‐1 overexpression could mediate EGF‐induced colon cancer cell proliferation via PI3K/Akt signalling pathway.^6^ Furthermore, it was reported that the activation of Src/STAT3/HO‐1/autophagy signalling was supposed to play a crucial role in protecting breast cancer cells from doxorubicin‐induced cytotoxicity.^7^ These contrasting observations have undoubtedly increased the significance of HO‐1 in the field of cancer biology. But there is rare research of the relationship between pharmorubicin resistance and autophagy in breast cancer.

Autophagy is regarded as the cell degradation of unnecessary or dysfunctional cell components, containing macromolecular compounds or cellular organelles, through the action of autophagosome. In normal cells, low level of autophagy could enhance recycling of cellular survival by depredating senescent organelles^2^ whereas in tumour cells, the role of autophagy is more complex.^8^ Ning et al found out that autophagy played a role in breast cancer resistance to chemotherapy, endocrine therapy and trastuzumab treatment.^2^ In breast cancers cells, the phosphatidylinositol 3‐kinase (PI3K)/Akt pathway plays a central role in cell growth, survival, proliferation and autophagy.^9^ Some researchers showed that deregulated activity of PI3K/Akt pathway might lead to uncontrolled cell growth, survival, migration and invasion for breast cancer cells contributing to tumour formation.^10^, ^11^ But not only that, fascine was proved to play a role in the chemotherapeutic resistance of breast cancer cells predominantly by the PI3K/Akt pathway.^12^ These researches showed that the PI3K/Akt pathway is closely related to the growth, chemotherapeutic resistance and autophagy of breast cancer cells.

This study would like to investigate the influence of HO‐1 on the resistance of pharmorubicin in breast cancer cells, and the effect of HO‐1 on cell survival and autophagy in pharmorubicin‐resistant cells. Furthermore, we researched the connection between PI3K/Akt signalling pathway and breast cancer cell autophagy.

## 2 MATERIALS AND METHODS

### 2.1 Cell culture

MDA‐MB‐231 was from ATCC cell bank (Manassas, VA), the cell line MCF‐7 was from cell bank of Chinese Academy of Sciences (Shanghai, China). MDA‐MB‐231 cells were grown in 90% DMEM medium supplemented with 10% FBS at 37°C in 5% CO_2_. MCF‐7 was grown in a high‐glucose (HyClone, Logan, Utah) Dulbecco's modified Eagle's medium (DMEM) containing 10% foetal bovine serum, 100 U/mL penicillin and 100 μg/mL streptomycin. The cells were incubated at 37°C and 5% CO_2_ in a humidified atmosphere.^13^ MDA‐MB‐231 and MCF‐7 are pharmorubicin‐sensitive human breast cancer cell lines.

### 2.2 Establishment of drug‐resistant cell lines

MDA‐MB‐231/EP1 was from ATCC cell bank and maintained in MEM‐EBSS containing 10% FBS at 37°C in 5% CO_2_. MCF‐7/EPI: After digested with 0.25% trypsin and centrifuged to collected parental MCF‐7 cells, 10 mL cell suspension with a density of 5 × 10^9^/mL was seeded in the culture bottle for 24 hours. The medium containing EPI (Pharmorubicin, Sangon Biotech, Shanghai, China) was added until the drug concentration reached 10 ng/mL then cultured for 48 hours. Digestion and subculture were conducted when cells growth recovered. Gradient concentration of EPI (10, 50, 100, 200, 500 ng/mL) was inducted. A 500 ng/mL drug‐resistant EPI cell line was obtained after 6 months’ cultivation as the drug‐resistant cell strain of breast cancer MCF‐7/EPI.^14^ MDA‐MB‐231/EP1 and MCF‐7/EPI are pharmorubicin‐resistant human breast cancer cell lines.

### 2.3 Materials

Pharmorubicin was purchased from Sigma (St. Louis, MO). LY294002 and chloroquine diphosphate (Chloroquine) were purchased from MCE (Monmouth Junction, NJ 08852, USA).

### 2.4 Cell transfection

PcDNA3.1 (‐)‐HO‐1 and siRNAs were from Gene Pharma (Shanghai, China). The cells were seeded into six‐well plates at 1 × 10^6^ cells per well at 37°C in 5% CO_2_ for 24 hours. Then, the cells were stably transfected with either pcDNA3.1 (‐)‐HO‐1, pcDNA3.1(‐)‐NC (empty vector) or siRNAs using Lipofectamine 3000 reagent (Invitrogen, Carlsbad, California, USA), as described by the manufacturer. After 3 hours, medium was added to the plates. After 2 days, the medium was replaced with the growth medium with selection reagent. The siRNAs were used to knock down HO‐1/AKt levels. For each experiment, a non‐targeting scramble siRNA was used as a negative control and two targeting siRNAs were used in the siRNA transfection. A known value of 20 μmol/L siRNAs and 5 μL of Lipofectamine 3000 (ThermoFisher Scientific, Grand Island, New York, USA) were diluted in 250 μL of serum‐free DMEM, separately, and incubated for 5 minutes at room temperature. Diluted siRNA was mixed with diluted Lipofectamine 3000 and incubated at room temperature for 20 minutes. Then, the complex of siRNA and Lipofectamine 3000 was added into cells, and culture was maintained for 72 hours at 37°C, 5% CO_2_ in a humidified incubator. At the end of transfection, cells were washed once with fresh medium before further experimentation.^15^, ^16^ The experiment group was performed as following: si‐HO‐1 and si‐HO‐1‐NC (non‐targeting); pcDNA3.1 (‐)‐HO‐1 and pcDNA3.1 (‐)‐NC (empty vector); chloroquine and NC (untreated); si‐Akt and si‐Akt‐NC (non‐targeting); LY294002 and NC (untreated).

### 2.5 Cell survival (MTT) assay

Cells were seeded into 96‐well plates at 5000 cells per well and were treated as indicated for 12‐48 hours depending on the experimental conditions. MTT (10 μL, 5 mg/mL) was added to per well and incubated for 4 hours. Finally, the medium from each well was replaced by 100 μL DMSO to dissolve the formazan before measurement on a microplate reader (Bio‐Rad Laboratories, Berkeley, California, USA) at 490 nm. The cell survival was normalized to the control group. The 50% growth inhibition concentration (IC_50_) of pharmorubicin at 12, 24 and 48 hours was examined in MDA‐MB‐231, MDA‐MB‐231/EP1, MCF‐7 and MCF‐7/EPI cells.

### 2.6 RT‐qPCR

To determine the mRNA expression of HO‐1, qRT‐PCR analysis was performed. Total RNA was extracted from cells by TRIzol^®^ (Invitrogen) and treated with DNase I (Invitrogen). cDNA was synthesized from 1 μg total RNA, using random primers with a ReverTra Ace qPCR RT Kit (Hitachi, Toyobo, Japan). The primer sequences real‐time PCR was performed with 7500 Fast Real‐time PCR system (Applied Biosystems, Foster City, California, USA), using Power SYBR Green PCR Master Mix (Applied Biosystems). With β‐actin as the internal reference and primer sequences used are set at Table 1. 2^−ΔΔCt^ was used to quantify the relative expression levels of each group.

**Table 1:** PCR primer sequence

Gene name	Primer sequence (5′‐3′)
HO‐1 forward	5′‐TTCTATCACCCTCTGCCT‐3′
HO‐1 reverse	5′‐CCTCTTCACCTTCCCCAACA‐3′
β‐actin forward	5′‐CACCATTGGCAATGAGCGGTTC‐3′
β‐actin reverse	5′‐AGGTCTTTGCGGATGTCCACGT‐3′

### 2.7 Western blotting analysis

After washing with cold PBS, cells were homogenized in RIPA lysis buffer (Beyotime, Jiangsu, China). Total crude protein (60 mg per well) was separated on 10% SDS‐PAGE gels. Then, it was transferred to PVDF membranes (Millipore, MA, USA). After washing for 10 minutes with TBST (0.1% Tween‐20, TBS) three times, the membranes were placed into blocking buffer (TBST with 5% non‐fat milk) for 1 hour at room temperature. And then, the membranes were incubated with primary antibodies against HO‐1 (0.5 μg/mL, 3391‐100; BioVision, San Francisco Bay Area, California, USA), PI3K p85 antibody (1:1000, #4292; Cell Signaling Technology, Beverly, Mass, USA), Akt (1:1000, #9272; Cell Signaling Technology), p‐Akt (1:1000, #9271; Cell Signaling Technology), LC3 (0.5 μg/mL, ab48394; Abcam, London, UK), Beclin‐1 (0.5 μg/mL, ab62557; Abcam) and β‐actin (1:2000, ab8227; Abcam) overnight at 4°C. The next day, after washing for 15 minutes with TBST (0.1% Tween‐20, TBS) four times, membranes were incubated with goat anti‐rabbit IgG (1:5000, ab6721; Abcam) for 1 hour at 37°C. Finally, it was visualized by ECL‐PLUS reagents (Beyotime). β‐actin was used for normalization of protein expression.

### 2.8 Cell autophagy analysis

The autophagy of cells was detected by Cyto‐ID Autophagy Detection Kit (Enzo Life Sciences, NY, USA). LC3II‐positive punctate pattern was observed under confocal microscope (Carl Zeiss LSM 510 META Laser Confocal Microscope, Oberkochen, Germany). Numbers of autophagosomes were counted using the ImageJ program (Version1.48u; Bethesda, MD).

### 2.9 Statistical analyses

Statistical analysis was carried out by the two‐tailed Student's *t* test or one‐way ANOVA. All analyses were performed using GraphPad Prism 6.0 (Version 6, San Diego, California, USA). Results are showed as mean ± SEM of at least three independent experiments. All tests were two‐sided, and *P* values < 0.05 were considered to be statistically significant.

## 3 RESULTS

### 3.1 The cell viability of MDA‐MB‐231 and MCF‐7 cells decreased by pharmorubicin at different treatment time

**Figure 1:**
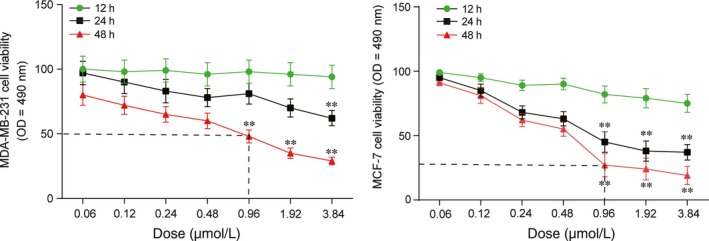
Pharmorubicin‐induced apoptosis in MDA‐MB‐231 and MCF‐7 cells affected by dose and treatment time. **P <* *0.01, compared with 12‐h group

The cell viability of MDA‐MB‐231 and MCF‐7 cells was examined by MTT assay after being treated with various concentrations of pharmorubicin (0.06‐3.84 μmol/L) for 12, 24 and 48 hours (Figure 1, *P *<* *0.01). It was found out that the cell viability of MDA‐MB‐231 and MCF‐7 was decreased significantly at 0.96 μmol/L in 48 hours group. Therefore, the cells which being treated with 0.96 μmol/L (IC_50_) pharmorubicin for 48 hours were used to the further experiments.

### 3.2 Pharmorubicin increased HO‐1 expression and autophagy in breast carcinoma cells

**Figure 2:**
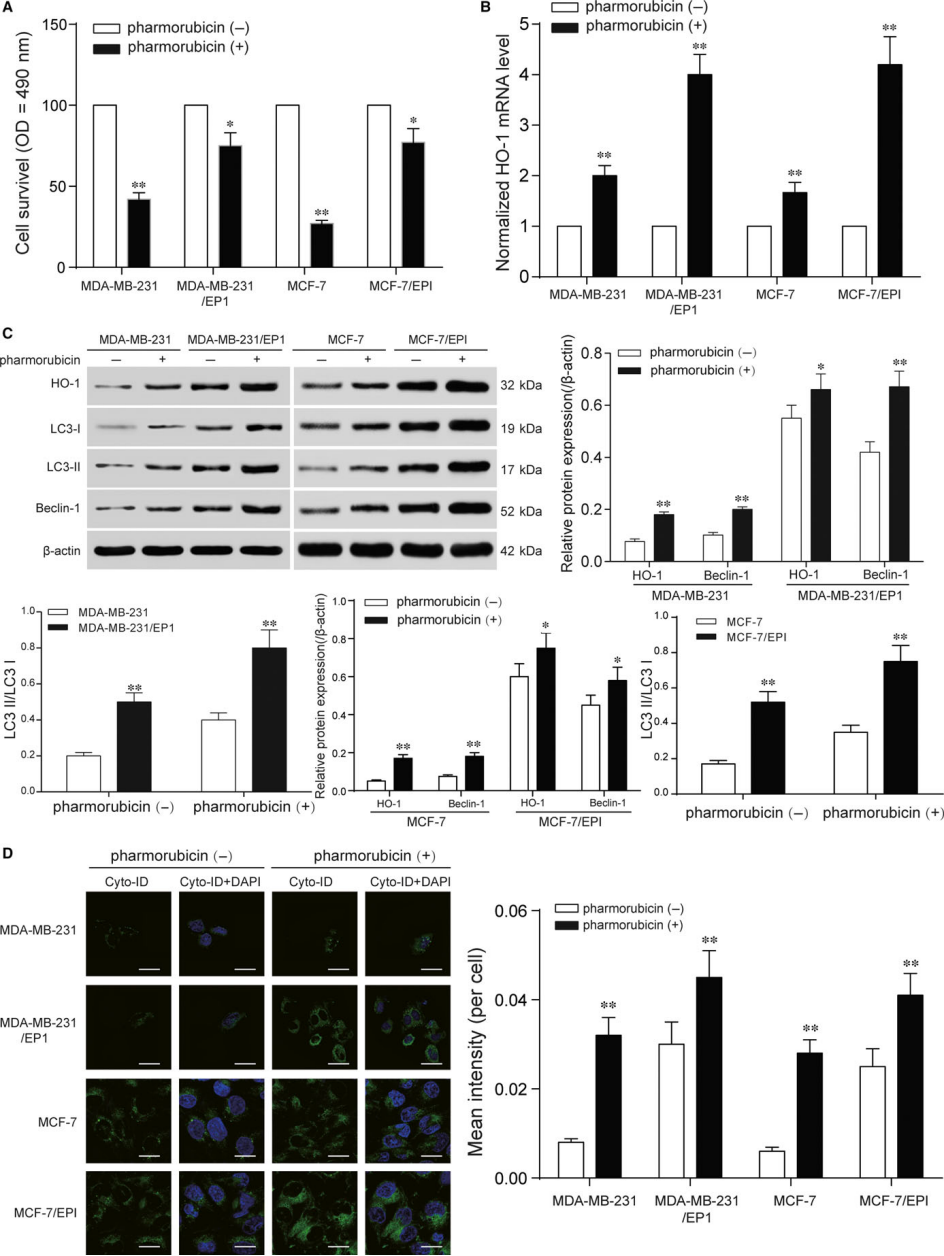
Induction of HO‐1 expression mediated pharmorubicin resistance in breast cancer cells. A, MTT assay revealed that the cell survival of MDA‐MB‐231/EP1 and MCF‐7/EPI was higher than MDA‐MB‐231 and MCF‐7 cells after being treated with pharmorubicin. B, The mRNA level in MDA‐MB‐231, MDA‐MB‐231/EP1, MCF‐7 and MCF‐7/EPI cells increased significantly after being treated with pharmorubicin. C, The expression of HO‐1, LC3‐II/LC3‐I and Beclin‐1 was up‐regulated in four group of cells after pharmorubicin treatment. D, The increase in pharmorubicin‐induced autophagy in four cell lines was observed by cell autophagy analysis, scale bar: 20 μm. *P <* *0.05, **P <* *0.01, compared with pharmorubicin (‐) or MDA‐MB‐231/MCF‐7 groups

To determine the sensitivity of chemoresistance in breast cancer cells, cell survival of four breast cancer cell lines, MDA‐MB‐231/EP1, MDA‐MB‐231, MCF‐7 and MCF‐7/EPI was tested by MTT assay. As shown in Figure 2A, a prominent decrease in cell survival was observed in MDA‐MB‐231 and MCF‐7 cells after 48‐hour pharmorubicin (0.96 μmol/L) treatment (*P *<* *0.05), while the cell survival in MDA‐MB‐231/EP1 and MCF‐7/EPI cells had a little decrease under the same pharmorubicin exposure conditions. After being treated with pharmorubicin, the mRNA and protein expression of HO‐1 was up‐regulated in the four group of cells (Figure 2B,C, *P *<* *0.01). Furthermore, the protein expression of Beclin‐1 and LC3‐II/LC3‐I was also up‐regulated in the four group of cells (Figure 2C, *P *<* *0.01) after pharmorubicin treatment. Cell autophagy assay revealed that the autophagy levels in pharmorubicin treatment group were higher than that in non‐pharmorubicin group (Figure 2D, *P *<* *0.01). The results showed that pharmorubicin increased HO‐1 expression and autophagy in breast carcinoma cells.

### 3.3 Inhibition of pharmorubicin‐induced autophagy decreased cell viability

**Figure 3:**
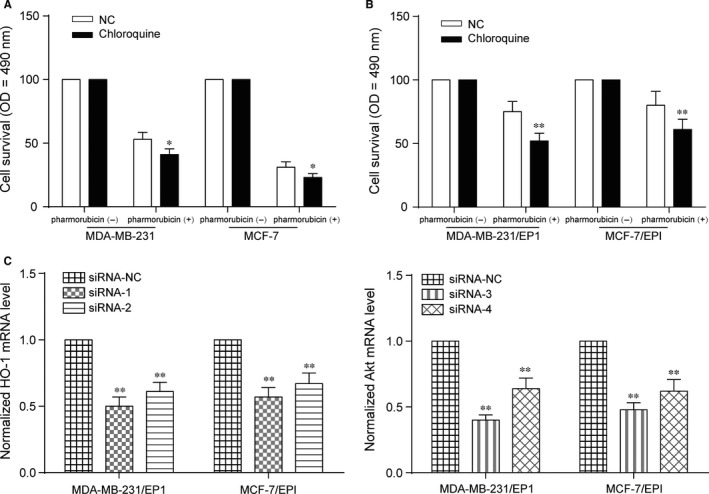
Inhibition of pharmorubicin‐induced autophagy down‐regulated cell viability and siRNAs selection. A, MTT assay revealed that the cell survival of chloroquine‐MDA‐MB‐231 and chloroquine‐MCF‐7 was decreased after being treated with pharmorubicin. B, The cell survival of chloroquine‐MDA‐MB‐231/EP1 and chloroquine‐MCF‐7/EPI was down‐regulated after pharmorubicin treatment. C, The mRNA expression level of HO‐1 and Akt was significantly down‐regulated with the transfection of siRNAs. *P <* *0.05, **P <* *0.01, compared with NC group

Chloroquine is an antimalarial drug that currently approved by Food and Drug Administration to treat rheumatoid arthritis and other autoimmune diseases as an autophagy inhibitor.^17^ To study the relationship between autophagy and chemoresistance, MDA‐MB‐231, MDA‐MB‐231/EP1, MCF‐7 and MCF‐7/EPI cells were treated with 10 μmol/L chloroquine for 48 hours, and then, cell survival of the cells after 0.96 μmol/L pharmorubicin treatment was detected by MTT assay. The cell survival of MDA‐MB‐231 and MCF‐7 in chloroquine group was lower than that in NC group after pharmorubicin treatment (Figure 3A, *P *<* *0.05). Similarly, the cell survival of MDA‐MB‐231/EP1 and MCF‐7/EPI was also decreased in chloroquine group after pharmorubicin treatment (Figure 3B, *P *<* *0.05). It was revealed that the suppression of autophagy could down‐regulate cell viability of breast cancer cells. In order to screen the siRNA, a non‐targeting siRNA and two targeting siRNAs were transfected into the cells. SiRNA‐1 had a better knockdown effectiveness on HO‐1 while siRNA‐3 had a better knock‐down effectiveness on Akt through detecting the mRNA expression level (Figure 3C, *P *<* *0.05). SiRNA‐1 was selected as si‐HO‐1, and siRNA‐3 was selected as si‐Akt in the subsequent experiments.

### 3.4 Pharmorubicin‐induced autophagy was regulated by HO‐1 in breast carcinoma cells

**Figure 4:**
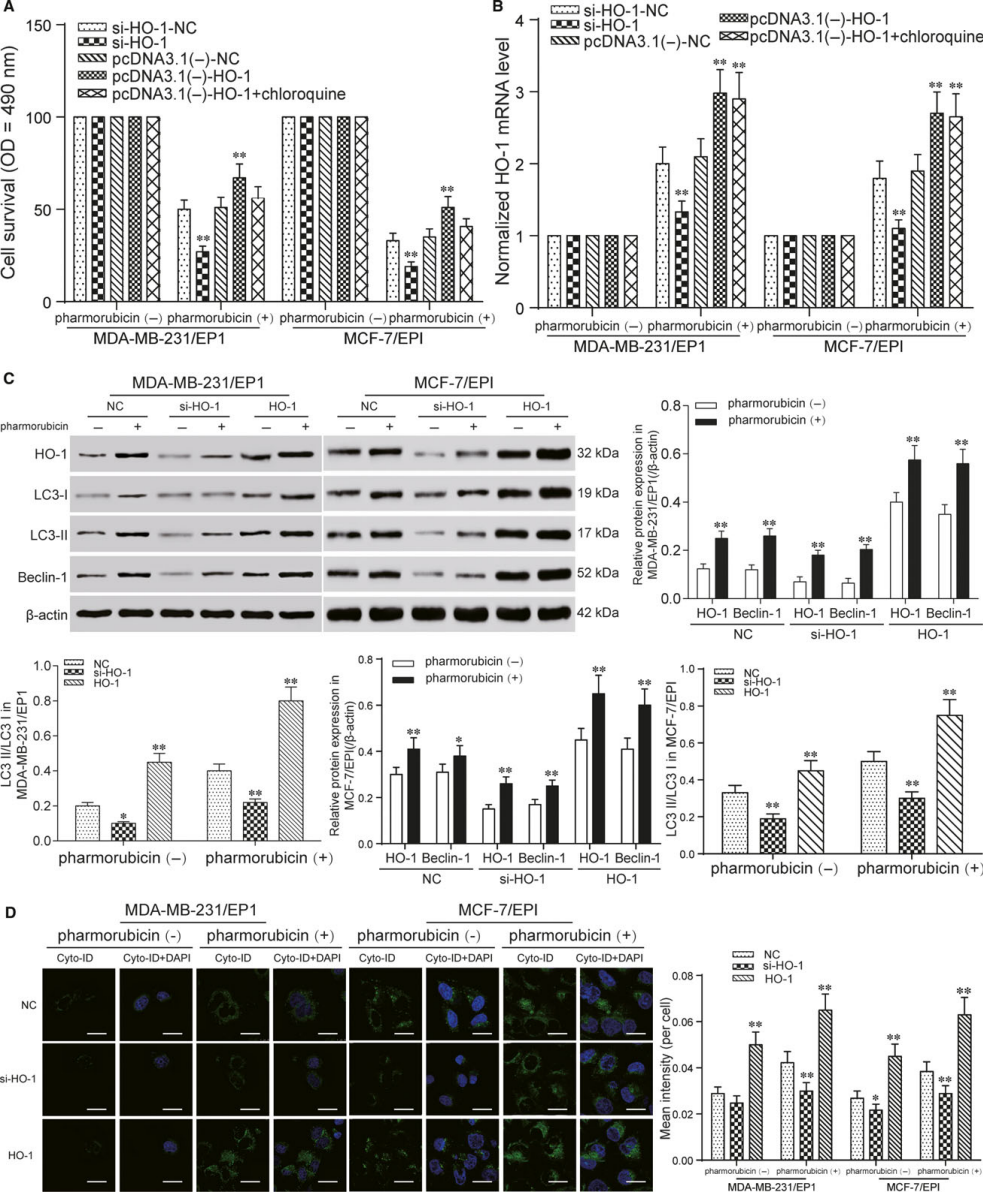
Pharmorubicin‐induced autophagy was regulated by HO‐1 in breast carcinoma cells. A, The cell survival of MDA‐MB‐231/EP1 and MCF‐7/EPI cells was detected by MTT. The results showed that the cell survival in si‐HO‐1 group decreased after pharmorubicin treatment. And in pcDNA3.1 (‐)‐HO‐1 group, the cell survival increased. B, QRT‐PCR detected the HO‐1 mRNA level in MDA‐MB‐231/EP1 and MCF‐7/EPI cells after being treated with pharmorubicin. The mRNA level of HO‐1 in si‐HO‐1 group was down‐regulated, and in pcDNA3.1 (‐)‐HO‐1 group, the mRNA expression was enhanced, compared with NC groups. C, The expression of HO‐1, LC3‐II/LC3‐I and Beclin‐1 in si‐HO‐1 group and HO‐1 group after pharmorubicin treatment was revealed by Western blot. The protein expression in si‐HO‐1 group was decreased, but increased in HO‐1 group. D, Cell autophagy analysis detected the autophagy of MDA‐MB‐231/EP1 and MCF‐7/EPI cells after being treated with pharmorubicin. The results illustrated the autophagy in HO‐1 group was up‐regulated, scale bar: 20 μm. *P <* *0.05, **P <* *0.01, compared with si‐HO‐1‐NC, pcDNA3.1 (‐)‐NC or NC group

In the further analysis, we tried to explore the downstream signalling following HO‐1 induction in mediating pharmorubicin resistance in breast cancer cells. MDA‐MB‐231/EP1 and MCF‐7/EPI cells were transfected with si‐HO‐1 and pcDNA3.1 (‐)‐HO‐1. In both of MDA‐MB‐231/EP1 and MCF‐7/EPI cells, the cell survival, mRNA and protein expression with the transfection of si‐HO‐1 were lower than that in si‐HO‐1‐NC group after being treated with pharmorubicin. In HO‐1 group (pcDNA3.1 (‐)‐HO‐1 group), the cell survival, mRNA and protein expression were higher than that in pcDNA3.1 (‐)‐NC group and the effect of pcDNA3.1 (‐)‐HO‐1 on cell survival could be eliminated by chloroquine (Figure 4A‐C, *P *<* *0.05). Furthermore, the protein expression of Beclin‐1 and LC3‐II/LC3‐I was decreased in si‐HO‐1 group and increased in HO‐1 group after being treated with pharmorubicin in MDA‐MB‐231/EP1 and MCF‐7/EPI cells (Figure 4C, *P *<* *0.01). Cell autophagy assay revealed that the autophagy of si‐HO‐1 group down‐regulated, but in HO‐1 group, the cell autophagy was up‐regulated obviously after pharmorubicin treatment (Figure 4D, *P *<* *0.05).

### 3.5 Akt was the upstream signal of HO‐1 in MDA‐MB‐231/EP1 and MCF‐7/EPI cells

**Figure 5:**
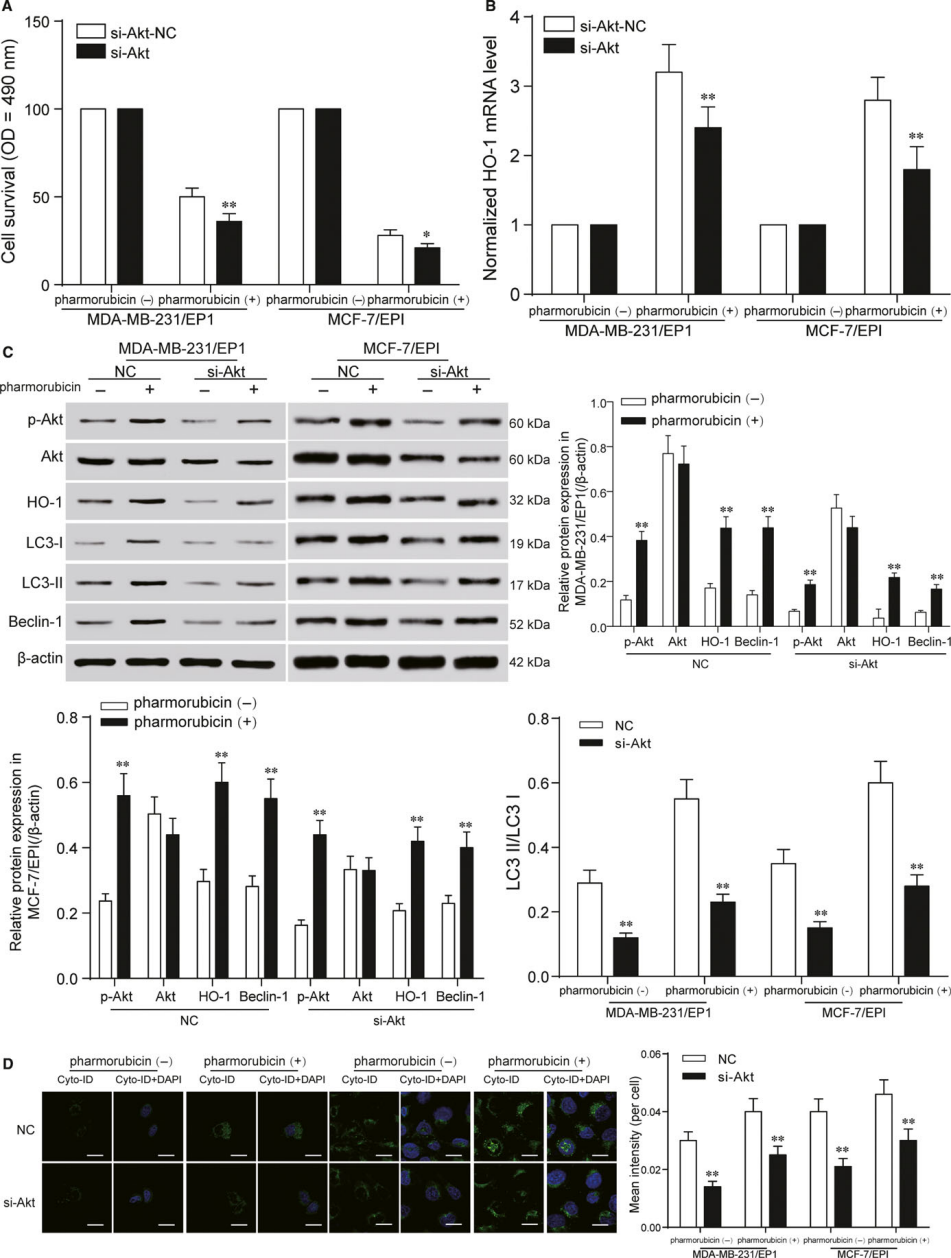
Akt was the upstream signal of HO‐1 inMDA‐MB‐231/EP1 and MCF‐7/EPI cells. A, The decrease in cell survival in si‐Akt group was showed in MTT assay. B, The result of qRT‐PCR revealed that the mRNA expression of HO‐1 in MDA‐MB‐231/EP1 and MCF‐7/EPI cells was down‐regulated after being transfected with si‐Akt. C, Western blot tested the protein expression of p‐Akt, HO‐1, Beclin‐1, LC3‐II/LC3‐I in si‐Akt group. The results showed that the protein expression in si‐Akt group was lower than that in NC group. D, The cell autophagy of MDA‐MB‐231/EP1 and MCF‐7/EPI cells in si‐Akt group was down‐regulated compared with NC group, scale bar: 20 μm. *P <* *0.05, **P <* *0.01, compared with si‐Akt‐NC, pharmorubicin (‐) or NC group

After HO‐1‐dependent autophagy in mediating the cytoprotective effect was revealed, we next focused on investigating the upstream signal events leading to HO‐1 induction in pharmorubicin‐induced responses. MDA‐MB‐231/EP1 and MCF‐7/EPI cells were transfected with si‐Akt to explore the effect of Akt on HO‐1 expression and pharmorubicin‐induced cell autophagy. After being treated with 0.96 μmol/L pharmorubicin in si‐Akt group, the decrease in cell survival was found by MTT assay (Figure 5A, *P *<* *0.05); qRT‐PCR showed the mRNA level of HO‐1 in si‐Akt group was down‐regulated (Figure 5B, *P *<* *0.01). The protein expression of p‐Akt, HO‐1, LC3‐II/LC3‐I and Beclin‐1 in si‐Akt group was lower than that in NC group under pharmorubicin exposure, and there was a same change trend in the cells with no pharmorubicin treatment (Figure 5C, *P *<* *0.01); Cell autophagy analysis illustrated that the cell autophagy of si‐Akt group was dramatically reduced comparing to NC group (Figure 5D, *P *<* *0.01). In summary, Akt is the upstream signalling factor of HO‐1 expression both in pharmorubicin‐induced cell and in pharmorubicin‐untreated cell autophagy.

### 3.6 PI3K functioned as the upstream protein kinase responsible for Akt activation in MDA‐MB‐231/EP1 and MCF‐7/EPI cells

**Figure 6:**
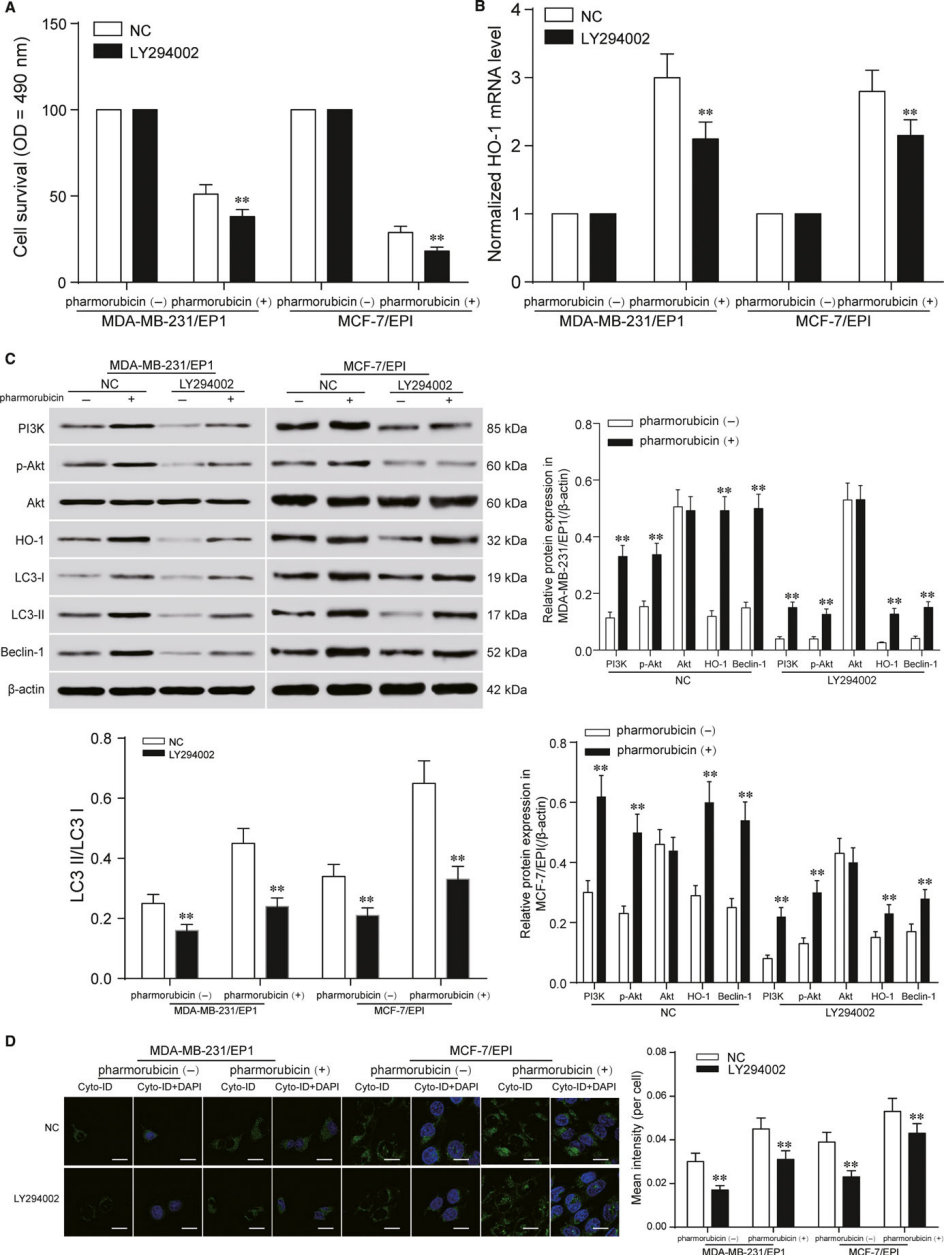
PI3K functioned as the upstream protein kinase responsible for Akt activation in MDA‐MB‐231/EP1 and MCF‐7/EPI cells. A, The cell survival of MDA‐MB‐231/EP1 and MCF‐7/EPI cells in LY294002 group was detected by MTT. The results showed that the cell survival in LY294002 group decreased in MDA‐MB‐231/EP1 and MCF‐7/EPI cells. B, qRT‐PCR detected the HO‐1 mRNA level in MDA‐MB‐231/EP1 and MCF‐7/EPI cells after being treated with pharmorubicin. The mRNA level of HO‐1 in LY294002 group was down‐regulated, compared with NC group. C, The expression of PI3K, Akt, p‐Akt, HO‐1, LC3‐II/LC3‐I and Beclin‐1 in LY294002 group after pharmorubicin treatment were examined by Western blot. The protein expression in LY294002 group was decreased. D, Cell autophagy assay detected the autophagy of MDA‐MB‐231/EP1 and MCF‐7/EPI cells after being treated with pharmorubicin. The result illustrated the autophagy in LY294002 group was down‐regulated, scale bar: 20 μm. *P <* *0.05, **P <* *0.01, compared with pharmorubicin (‐) or NC group

LY294002 is a synthetic inhibitor of PI3K based on the structure of quercetin, and it inhibits PI3K by binding reversibly to the ATP site of PI3K.^18^ After MDA‐MB‐231/EP1 and MCF‐7/EPI cells were treated with 10 μmol/L LY294002 under 0.96 μmol/L pharmorubicin for 48 hours, the cell survival, HO‐1 mRNA and protein expression in LY294002 group declined compared with NC group (Figure 6A‐C, *P *<* *0.05). Furthermore, the inhibition of PI3K could down‐regulate the protein expression of p‐Akt, LC3‐II/LC3‐I and Beclin‐1 (Figure 6C, *P *<* *0.05). Cell autophagy analysis showed the autophagy in LY294002 group was down‐regulated compared with NC group (Figure 6D, *P *<* *0.05).

## 4 DISCUSSION

Breast cancer is a dominant cause of cancer‐associated death in women worldwide.^3^ Although surgery, systemic therapy and radiotherapy have been applied for the clinical treatment of breast cancer, these approaches are limited to some extent, because of potential complications or drug resistance.^19^ Thus, growing tumour cell sensitivity to chemotherapeutic drugs is an important goal towards enhancing the therapeutic efficacy of breast cancer, and regulation of tumour‐specific signalling is supposed to provide a complementary and different way.^7^ In order to explore the molecular functions of autophagy, cell survival as well as drug resistance in breast cancer, this study examined the ability of HO‐1 induction to cell survival and pharmorubicin resistance of breast cancer cells and delineated the connection between PI3K/Akt signalling and autophagy in breast cancer cells to offer evidence for the improvement of breast cancer‐targeted therapies.

As one of the crucial regulators for autophagy, the role of HO‐1 in drug‐induced autophagy of breast cancer cells seems more complicated.^7^ Some reports demonstrated the mechanism of HO‐1 in inhibiting breast tumour cell proliferation and migration in response to other anti‐cancer drugs. Further, Tan et al suggested that HO‐1 induction enhances doxorubicin resistance of breast cancer cells via promoting autophagy,^7^ while others confirmed HO‐1 induction shows a cytoprotective autophagy in breast cancer cells.^7^ In our study, the results showed that the autophagy and pharmorubicin resistance of breast cancer cells were induced by promoting HO‐1 expression.

Autophagy is known to be connected with cancer in two contrasting mechanisms in that it is either a tumour inhibitor or a tumour protector.^2^, ^20^ The dual role of autophagy in cancer is augmented by conflicting reports about the effect of autophagy on chemotherapeutic treatment.^7^ Ning et al and Yu et al found out that the suppression of autophagy might promote the drug resistance of breast cancer cell.^2^, ^21^ However, some reports showed that autophagy is induced in cancer cells as a survival strategy against these drugs.^22^ For example, Zhang et al proposed that TRPC5‐induced autophagy promoted drug resistance in breast carcinoma via CaMKKβ/AMPKα/mTOR pathway.^23^ Similarly, the results in our study showed that the pharmorubicin resistance was up‐regulated after promoting autophagy in breast cancer cells.

Because of these facts, it was confirmed that regulation of autophagy by HO‐1 appears to be one of the most important functions to regulate the drug resistance of breast cancer cells. PI3K/Akt signalling pathway is one of the major upstream cellular signals in breast cancer cells.^2^ Zhong et al found that endoplasmic reticulum stress activation could promote breast cancer cell autophagy and apoptosis and enhance chemosensitivity of MCF‐7 cells by inhibiting the PI3K/AKT/mTOR signalling pathway.^19^ In current study, we found the HO‐1 up‐regulated by p‐Akt promoted autophagy and pharmorubicin resistance. The result suggested that the inhibition of PI3K/Akt signalling pathway might contribute to the pharmorubicin resistance in breast cancers cells.

In this study, we verified that HO‐1 induction could mediate pharmorubicin resistance by promoting autophagy via PI3K/Akt pathway in breast cancer cells. The molecular mechanism of pharmorubicin resistance mediated by HO‐1 in breast cancer cells was illustrated. But the mechanism of invasion and migration regulated by HO‐1 in breast cancer cells was not researched currently. In the further researches, the effects of HO‐1 on invasion and migration of breast cancer cells will be investigated and the animal experiments for exploring the pharmacokinetics in vivo are necessary.

In conclusion, autophagy and the expression of HO‐1 were both up‐regulated in MDA‐MB‐231, MDA‐MB‐231/EP1, MCF‐7 and MCF‐7/EPI cells. Furthermore, down‐regulation of HO‐1 expression resulted in the decline of autophagy so that the pharmorubicin resistance was down‐regulated by PI3K/Akt signalling in breast cancer cells. HO‐1 could be developed as a novel potential therapeutic target to attenuate pharmorubicin resistance, leading to the improvement of clinical use of pharmorubicin in breast cancer cells.

## DISCLOSURE

Ethics approval and consent to participate: N/A; Consent for publication: This manuscript has been approved by all authors for publication; Availability of data and material: N/A.

## CONFLICT OF INTERESTS

The authors confirm that there are no conflicts of interest.

## AUTHORS’ CONTRIBUTIONS

Lei Pei, Yirong Kong, Zongling Wang performed contributing to the conception and design; Changfeng Shao, Xiao Yue, Na Zhang involved in analysing and interpreting data; Lei Pei, Yirong Kong, Na Zhang performed drafting the article; Changfeng Shao, Xiao Yue, Zongling Wang, Na Zhang performed revising it critically for important intellectual content; All authors involved in approving the final version to be published.
